# UK ethnic minority healthcare workers’ perspectives on COVID-19 vaccine hesitancy in the UK ethnic minority community: A qualitative study

**DOI:** 10.3389/fpsyg.2022.908917

**Published:** 2022-08-03

**Authors:** Dominic Sagoe, Charles Ogunbode, Philomena Antwi, Birthe Loa Knizek, Zahrah Awaleh, Ophelia Dadzie

**Affiliations:** ^1^Department of Psychosocial Science, University of Bergen, Bergen, Norway; ^2^School of Psychology, University of Nottingham, Nottingham, United Kingdom; ^3^Department of Health and Exercise, Kristiania University College, Bergen, Norway; ^4^Department of Mental Health, Norwegian University of Science and Technology, Trondheim, Norway; ^5^Chaplaincy, Royal Free London NHS Foundation Trust, London, United Kingdom; ^6^Department of Dermatology, Hillingdon Hospitals NHS Foundation Trust, London, United Kingdom; ^7^Department of Cellular Pathology, Northwest London Pathology, Imperial College London NHS Trust, London, United Kingdom

**Keywords:** COVID-19, ethnicity, healthcare workers, qualitative research, vaccine hesitancy, ethnic minority, UKEM

## Abstract

**Background:**

The experiences of UK ethnic minority (UKEM) healthcare workers are crucial to ameliorating the disproportionate COVID-19 infection rate and outcomes in the UKEM community. We conducted a qualitative study on UKEM healthcare workers’ perspectives on COVID-19 vaccine hesitancy (CVH) in the UKEM community.

**Methods:**

Participants were 15 UKEM healthcare workers (11 females; age range: 26–58 [43.3 ± 9.4] years). Data were collected using individual and joint interviews, and a focus group, and analyzed using thematic analysis.

**Results:**

We generated three themes: heterogeneity (two subthemes), mistrust (six subthemes), and mitigating (six subthemes). Therein, participants distinguished CVH in the UKEM community in educational attainment and ethnicity. They pointed to the role of mistrust in CVH in the UKEM community. They opined that the mistrust underlying CVH in the UKEM community is rooted in history and religion, conspiracy theories, the speedy development and novelty of the vaccines, post-vaccination complications/side effects, false positive test results, and social media and social support/influence. Participants recommended that interventions targeted at mitigating CVH in the UKEM community need to, in a non-judgmental way, tackle dis/misinformation and provide education, and incorporate UKEM healthcare worker endorsement. They also suggested such interventions be community-oriented, enhance the convenience of vaccination centers and the possibility of vaccine choice, and appreciate that overcoming CVH and accepting vaccination is a gradual process involving personal assessment of risks and benefits.

**Conclusion:**

CVH in the UKEM community is a multifaceted phenomenon requiring multicomponent interventions.

## Introduction

The main strategy deployed by many countries to mitigate the effects of the coronavirus disease 2019 (COVID-19) has been the widespread use of vaccines. However, the success of this strategy depends on widespread vaccine uptake. COVID-19 vaccine hesitancy (CVH), defined here as delay in accessing or refusal of available COVID-19 vaccines, is a global problem and a major threat to COVID-19 vaccination programs (Biswas et al., 2021; Sallam, 2021; Sallam et al., 2022). Globally, the preponderance of literature on COVID-19 vaccination estimates a general population acceptance rate of 70%, whereas UK acceptance rates are estimated at 79, 83, 72, 64, and 72% for April, May, June, July, and September/October 2020 respectively (Sallam, 2021).

Theoretical models of behavioral initiation and change such as the integrative model of behavioral prediction (Fishbein, 2000, 2008), the socio-ecological model (Brofenbreener, 1979), and the vaccine hesitancy model (Peretti-Watel et al., 2015) emphasize the significance of personal (e.g., knowledge and values), interpersonal (e.g., family network), community (e.g., ethnic and religious communities), institutional (e.g., health and vaccine delivery institutions), and public policy (i.e., local, regional, and national policy and strategy) factors in vaccine hesitancy (Larson et al., 2014; Dubé et al., 2018; Fall et al., 2018) and CVH in particular (Eberhardt and Ling, 2021a; Husain et al., 2021; Zimand-Sheiner et al., 2021; Cambon et al., 2022).

Despite the above evidence, the relative novelty of COVID-19 vaccination underlines the importance of more research examining factors associated with CVH (Acharya et al., 2021). Such research is crucial for the development of interventions targeted at mitigating CVH and boosting COVID-19 vaccine uptake (Lazarus et al., 2021; Cambon et al., 2022). At the community level, CVH research in specific populations may facilitate the development and deployment of targeted interventions (Jaiswal et al., 2020; Bogart et al., 2021; Murphy et al., 2021).

There is extant evidence showing that UK ethnic minority (UKEM) groups have a higher prevalence and mortality from COVID-19 (Aldridge et al., 2020; Niedzwiedz et al., 2020; Mathur et al., 2021). However, a scoping review of CVH literature in affluent countries suggests that not being of white ethnicity is associated with increased CVH (Aw et al., 2021). Indeed, a systematic review of factors influencing COVID-19 vaccination uptake in minority ethnic groups in the UK indicates that UKEM status, compared to White British status, is associated with higher CVH (Kamal et al., 2021). With persons of UKEM background having higher prevalence and mortality from COVID-19 as evident above, understanding the motivations for CVH in the UKEM community is important to guide the development and deployment of interventions targeted at mitigating CVH and boosting COVID-19 vaccine uptake.

It is notable that healthcare workers are regarded as the most trusted source of information on vaccines, and they are in a privileged position to appreciate the concerns of hesitant persons, respond to their apprehensions, and provide education on the benefits of vaccination (European Centre for Disease Prevention and Control, 2015a). As such, healthcare workers are at the frontline of the COVID-19 battle and are also crucial to the CVH mitigation effort in the community (World Health Organization and United Nations Children's Fund, 2021). Particularly, UKEM healthcare workers are at the intersection of the COVID-19 pandemic battle frontline and the UKEM community. In this sentinel location, the perspectives of UKEM healthcare workers are crucial to ameliorating the disproportionate COVID-19 infection and outcomes in the UKEM community.

An important methodical approach for investigating the COVID-19 experiences of UKEM healthcare workers is qualitative research methods such as focus groups and interviews as they facilitate discussion between researchers and participants thereby providing deeper insight into participants’ experiences (Tremblay et al., 2021; World Health Organization, 2021). Thus, we conducted a qualitative study on UKEM healthcare workers’ experiences of CVH in the UKEM community. Accordingly, the overarching research question guiding the present study is: what are the perspectives of UKEM healthcare workers on CVH in the UKEM community?

## Materials and methods

### Design

The present study was conducted as part of a larger mixed-method study on the phenomenology and correlates of COVID-19 in UKEM healthcare workers. We used a qualitative design comprising individual interviews, a joint interview (interviewing two participants together), and a focus group. Although the interviews and focus group were both useful for exploring participants’ perspectives on vaccine hesitancy, the joint interviews and focus group had additional advantages of highlighting areas of contrast (Kidd and Parshall, 2000; Sagoe, 2012; Polak and Green, 2016). Thus, apart from yielding different sets of data, the mixed qualitative design also has a method triangulation benefit (Carter et al., 2014).

### Participants and procedure

Participants were 15 UKEM healthcare workers (females: *n* = 11) aged 26 to 58 (*M* = 43.3, SD = 9.4) years. All are of non-European ancestry. They worked in various geographical regions as doctors (*n* = 6), nurses (*n* = 6), an administrator (*n* = 1), a chaplain (*n* = 1), and a psychologist (*n* = 1). Work experience ranged from 3 to 360 (*M* = 135.2, SD = 104.1) months. In-depth interviews were conducted with 11 participants, a joint interview with two (focus group dropout: *n* = 3) participants, and a focus group with three (dropout: *n* = 2) participants. Other participant characteristics are presented in Tables 1, 2.

**Table 1:** Participant description.

Participant	Pseudonym	Gender	Age	Ethnicity	Country of birth	Profession
Interviewee 1	INT1	Female	51	Asian/Asian British	UK	Administrator
Interviewee 2	INT2	Male	26	Asian/Asian British	Myanmar	Doctor
Interviewee 3	INT3	Female	47	Black/African/Caribbean/Black British	Nigeria	Nurse
Interviewee 4	INT4	Male	37	Black/African/Caribbean/Black British	Nigeria	Doctor
Interviewee 5	INT5	Male	46	Asian/Asian British	India	Nurse
Interviewee 6	INT6	Female	33	Black/African/Caribbean/Black British	Nigeria	Nurse
Interviewee 7	INT7	Female	46	Black/African/Caribbean/Black British	Somalia	Doctor
Interviewee 8	INT8	Female	50	Black/African/Caribbean/Black British	UK	Psychologist
Interviewee 9	INT9	Female	53	Asian/Asian British	Iran	Nurse
Interviewee 10	INT10	Male	45	Asian/Asian British	India	Nurse
Joint interviewee 1	JINT1	Female	47	Black/African/Caribbean/Black British	UK	Chaplain
Joint interviewee 2	JINT2	Female	48	Asian/Asian British	UK	Doctor
Focus group participant 1	FGP1	Female	33	Black/African/Caribbean/Black British	UK	Doctor
Focus group participant 2	FGP2	Female	58	Mixed/multiple ethnic groups	India	Doctor
Focus group participant 3	FGP3	Female	29	Asian/Asian British	Australia	Nurse

**Table 2:** Summary of participant characteristics.

	Overall (*N* = 15)		Individual interview (*n* = 10)		Joint interview (*n* = 2)		Focus group (*n* = 3)	
Variable	*n*	%	*n*	%	*n*	%	*n*	%
**Gender**								
Female	11	73.3	6	60.0	2	100	3	100
Male	4	26.7	4	40.0	0	0	0	0
**Country of birth**								
Australia	1	6.7	0	0.0	0	0.0	1	33.3
India	3	20.0	2	20.0	1	50.0	1	33.3
Iran	1	6.7	1	10.0	0	0.0	0	0.0
Myanmar	1	6.7	1	10.0	0	0.0	0	0.0
Nigeria	3	20.0	3	30.0	0	0.0	0	0.0
Somalia	1	6.7	0	0.0	1	50.0	0	0.0
UK	5	33.3	2	20.0	0	0.0	1	33.3
**Ethnicity**								
Asian/Asian British	7	46.7	5	50.0	1	50.0	1	33.3
Black/African/Caribbean/Black British	7	46.7	5	50.0	1	50.0	1	33.3
Mixed/multiple ethnic groups	1	6.7	0	0.0	0	0.0	1	33.3
**Relationship status**								
Single, never married	3	20.0	1	10.0	0	0.0	2	66.7
Married	9	60.0	8	80.0	1	50.0	0	0.0
Divorced	3	20.0	1	10.0	1	50.0	1	33.3
**Profession**								
Administrator	1	6.7	1	10.0	0	0.0	0	0.0
Chaplain	1	6.7	0	0.0	1	50.0	0	0.0
Doctor	6	40.0	3	30.0	1	50.0	2	66.7
Nurse	6	40.0	5	50.0	0	0.0	1	33.3
Psychologist	1	6.7	1	10.0	0	0.0	0	0.0
**Country of initial qualification**								
Australia	1	6.7	0	0.0	0	0.0	1	33.3
India	1	6.7	1	10.0	0	0.0	0	0.0
Iran	1	6.7	1	10.0	0	0.0	0	0.0
Myanmar	1	6.7	1	10.0	0	0.0	0	0.0
Nigeria	2	13.3	2	20.0	0	0.0	0	0.0
UK	9	60.0	5	50.0	2	100.0	2	66.7
**Dropout**								
Emergency situation	–	–	–	–	1	33.3	0	0.0
Failed access to meeting	–	–	–	–	1	33.3	0	0.0
Not reachable despite appointment	–	–	–	–	1	33.3	2	100.0
	**Range**	***M* (*SD*)**	**Range**	***M* (*SD*)**	**Ages**	***M* (*SD*)**	**Range**	***M* (*SD*)**
Age (years)	26–58	43.3 (9.4)	26–53	43.4 (8.7)	47, 48	47.5 (0.7)	29–58	43.4 (8.7)
Work experience (months)	3–360	135.2 (104.1)	15–360	163.5 (105.4)	72, 204	138.0 (93.3)	3–108	39.0 (59.8)

Percentages may not add up to 100 due to rounding.

The inclusion criterion was that participants were UKEM healthcare workers. We did not control for any other demographic and profession factor in recruitment. Participants were recruited by OD *via* convenience sampling through personal contacts, posts on social media pages of healthcare worker groups, and contacts with UKEM healthcare worker networks. OD had prior working relationships with three of the participants. Irrespective, all participants received detailed information about the study and the researchers prior to volunteering to participate.

Interviews and the focus group were conducted by DS and OD. OD has been a dermatology and dermatopathology consultant in the UK National Health Service (NHS) since 2010. OD’s clinical and research work (e.g., Dadzie et al., 2022) has focused on cutaneous diversity particularly in UKEM groups. DS is professor of psychology with extensive teaching and research experience in research methods (e.g., Sagoe, 2012). Our data collection team therefore blends competence in medicine, particularly in the UKEM community (OD), with qualitative research methods and psychology expertise (DS).

Data collection was conducted between October and November 2021 when vaccines were available to all adults aged 18 years and over in the UK. The interviews and focus group were conducted on Zoom (Zoom Video Communications, Inc.) and ranged in duration from 33 to 100 (*M* = 56.50, SD = 19.32) minutes. They were recorded for transcription. Data collection was undertaken at the convenience of participants’ homes or offices with only the interviewers and interviewees present. Data saturation was reached *via* the non-emergence of novel perspectives. Each participant was gifted a £30 Marks & Spencer voucher as compensation for taking part in the study. The study is fully compliant with the British Psychological Society’s code of human research ethics (Oates et al., 2021) and the declaration of Helsinki. Ethical approval was obtained from the Faculty Research Ethics Committee of the Faculty of Health and Life Sciences, De Montfort University (HLS FREC Ref: 3662). The study is presented according to the Consolidated Criteria for Reporting Qualitative Research (COREQ: Tong et al., 2007).

### Interview guide

A semi-structured interview guide was used in data collection. Participants provided demographic and work-related information. Two key questions were used in exploring participants’ perspectives on vaccine hesitancy in the UKEM community:

a. what do you think about COVID-19 vaccine hesitancy in the UKEM community?
b. do you think religion/religiosity has a role to play in covid-19 vaccine hesitancy in the UKEM community?

### Data analysis

We used descriptive statistics comprising frequencies, proportions, means and standard deviations to ascertain sample characteristics. Analysis of the qualitative data was conducted using thematic analysis (Braun and Clarke, 2006, 2012). The analysis was inductive. First, audio recordings were transcribed verbatim, and the first author read through the transcripts several times to gain an understanding of the data. Second, initial codes were generated across the data. Third, initial codes were collated into initial themes. Fourth, initial themes were reviewed by examining connections between them. Fifth, final themes were generated by clustering initial themes based on commonalities. Finally, the most salient quotes were selected as representation of final themes. The descriptive statistical analysis was conducted using SPSS version 25 (IBM Corp.) and the qualitative analysis using NVivo version 12 (QSR International).

## Results and discussion

All participants voluntarily disclosed they had taken their COVID-19 vaccines. Three themes were generated from the thematic analysis: heterogeneity (two subthemes); mistrust (six subthemes); and mitigating (six subthemes). An overview of the themes is presented in Table 3 and elaborated below.

**Table 3:** CVH themes and codes/subthemes.

Themes	Codes/subthemes
1. Heterogeneity	a. Educational heterogeneity
	b. Ethnic heterogeneity
2. Mistrust	a. Historical and religious mistrust
	b. Conspiracy theories
	c. Precipitancy and novelty
	d. Complications/side effects
	e. False positive test results
	f. Social media and social support/influence
3. Mitigating	a. Combating dis/misinformation and educating
	b. Non-judgmental approach
	c. Healthcare worker endorsement
	d. Community-oriented approach
	e. Convenience and self-determination
	f. Gradual acceptance *via* risk–benefit self-assessment

### Heterogeneity

Vaccine hesitancy is a multifaceted phenomenon (Jarrett et al., 2015) and vaccine-hesitant persons are a heterogeneous population (European Centre for Disease Prevention and Control, 2015a). Similarly, it emerged that COVID-19 vaccine-hesitant UKEM persons are a heterogeneous group in line with evidence from a systematic review of correlates of CVH in UKEM groups (Kamal et al., 2021). Participants elaborated heterogeneity under the subthemes: educational heterogeneity and ethnic heterogeneity.

#### Educational heterogeneity

Participants highlighted the important role of educational attainment in CVH in the UKEM community. Some indicated that persons in the UKEM community who are vaccine-hesitant can be differentiated by their educational level. The following quote illustrates this position:

The issue [CVH] would be a difference in minorities who are much highly educated and those who are not that much educated. I think there would be a difference in the perception in those groups. Because, sometimes, [for] most of our ethnic minority healthcare workers, and sometimes most of the ethnic minority from the other professions, the educated group are very much thankful to [be] vaccinated…but [not] in some communities of minority ethnic origins because of the low education background (Interviewee [INT] 2, doctor).

INT2 opines that highly educated persons of UKEM background, such as healthcare workers and other professionals, tend to have less CVH whereas those of lower educational attainment tend to have higher CVH. This perspective is supported by evidence from a narrative literature review showing a frequent association of lower education with high CVH in affluent nations (Aw et al., 2021), and evidence from the UK showing that higher CVH is associated with lower educational attainment (Allington et al., 2021; Robertson et al., 2021). However, one participant discounted the above lower educational attainment explanation:

I think people [who are vaccine-hesitant] chose to listen to the rubbish because I don’t believe that the BAME [- black, Asian and minority ethnic - community] isn’t educated. I believe the BAME [community] is maybe even more educated. Normally, the excuses: oh, uneducated, they don’t understand…But you can’t even say that no more about the BAME people because they are very well educated. And in fact, I’ve noticed that people with the Afro-Caribbean descent are very pushed by their families to be educated and so on. So yeah, we can’t hide behind that [educational attainment] excuse no more (INT1, administrator).

This participant explains that since the UKEM community places great emphasis on and promotes education and academic achievement, acceptance of dis/misinformation rather than lower educational attainment, accounts for CVH in the UKEM community. Despite this assertion, there is indication that more effort is required, particularly in UK higher education, to narrow the UKEM educational attainment gap (Universities UK National Union of Students, 2019; Jankowski, 2020). This is important for mitigating CVH (Allington et al., 2021; Robertson et al., 2021) and vaccine hesitancy in general (Dubé et al., 2015; De Figueiredo et al., 2020).

#### Ethnic heterogeneity

As noted previously, non-white ethnicity is often associated with high CVH in affluent countries (Aw et al., 2021). The higher rate of CVH in the UKEM community was explained by some participants in terms of the perceived disregard of the UKEM community in the vaccine trials and development. In the focus group, one participant described her observation as the only person of UKEM background on the COVID-19 vaccine trials she was involved in as a researcher:

So like there was quite a stark lack of BAME, ethnic minority, participants in these [COVID-19 vaccine] trials. And I guess there’s loads of reasons for that. And then when we did have the vaccine approved and stuff and then obviously, you know, we found the rates of vaccine hesitancy was a lot higher in ethnic minority communities (Focus group participant [FGP] 1, doctor).

FGP1 observes the lack of UKEM participants in the COVID-19 trials and suggests that this may account for the high CVH in the UKEM community. This perspective reflects evidence from a similar UK study (Woodhead et al., 2021). It is also consistent with findings from a systematic review of literature on CVH in UKEM groups where the lack of representation of UKEM participants in the vaccine trials was linked to high CVH (Kamal et al., 2021). Similar to educational heterogeneity, another participant described an observation of ethnic heterogeneity in CVH within the UKEM community:

I think the Pakistani Indian people have probably moved faster and got to wrap their heads around the attitude of COVID and whether they should or should not have the vaccine. I know every member of my Asian family and Asian networks that are not related to me have now had the vaccine. They didn’t, they were very anti-vaccine to start with but now they have all had it. But with my Afro-Caribbean Jamaican people, they still have not had the vaccine (INT1, administrator).

The participant narrates above her observation that relative to her Afro-Caribbean relatives and friends, her Asian relatives and friends had an easier navigation of CVH. This observation is supported by evidence from a systematic metasynthesis of literature on correlates of CVH in UKEM groups where 12 of 21 studies reported higher CVH in Black groups in comparison to other minority ethnic groups (Kamal et al., 2021). Indeed, empirical evidence from the UK shows that in comparison to White British/Irish groups, Black groups are 13.4 times more likely to be COVID-19 vaccine-hesitant versus 2.5 times for Pakistani/Bangladeshi groups (Robertson et al., 2021). Similar ethnic differences in CVH have been reported among UK healthcare workers (Woolf et al., 2021).

### Mistrust

Trust, a connotation of the belief in the reliability and good intentions of other persons, institutions, or processes is crucial for cooperation and coordinated policy in resolving the COVID-19 pandemic (Balog-Way and McComas, 2020; Cairney and Wellstead, 2021). Mistrust of government and health authorities is a key explanation for vaccine hesitancy (European Centre for Disease Prevention and Control, 2015a,b; Karafillakis et al., 2016; Universities UK National Union of Students, 2019) and CVH in particular (Freeman et al., 2020; Aw et al., 2021; Bogart et al., 2021; Deal et al., 2021; Lockyer et al., 2021; Murphy et al., 2021). In the present study, participants described the role of mistrust in CVH in the UKEM community under the following subthemes: historical and religious mistrust, conspiracy theories, precipitancy and novelty, complications/side effects, false positive test results, and social media and social support/influence.

#### Historical and religious mistrust

Historical evidence of unethical medical practice in the UKEM community (Acharya et al., 2021) was denoted as a key factor in CVH. One participant explained:

I think it’s [hesitancy] always been a traditional theme within the BAME community…And this is a historical thing. We know about cases of slaves as basically Guinea pigs for [the Tuskegee] syphilis [study]. And even pregnant ladies being used. For example, the Sims speculum was actually developed from pregnant slaves. And they were really subjected to a lot of horrific examinations that would not be deemed acceptable in this day and age. So I think that there’s a history behind this [CVH] and there’s a reason why the BAME community [is] more hesitant towards any new intervention (INT4, doctor).

In the above quote, the participant cites notable instances of historical medical malpractice as a background to CVH in the UKEM community. This perspective is supported by the literature on vaccine hesitancy in general (Aw et al., 2021), and literature on CVH in the UKEM community in particular (Acharya et al., 2021; Eberhardt and Ling, 2021a; Woodhead et al., 2021). For instance, as in the US Black community (Bogart et al., 2021), a systematic metasynthesis of literature on CVH in UKEM groups shows that Black peoples’ mistrust of vaccines is often rooted in their historical medical maltreatment exemplified in the Tuskegee syphilis study (Kamal et al., 2021).

Also, religiosity has been implicated in vaccine hesitancy in general (European Centre for Disease Prevention and Control, 2015a,b). As a key feature of the UKEM community, several participants mentioned the role of religion in CVH. Majority of participants pointed to religion without questioning or prompting. One participant explained that:

It [CVH] can also come from faith and ideas about religiosity…What’s behind, you know? And is this demonic?…Now for the Muslim faith, I think you know there are people who haven’t taken the vaccine because they believe there’s pork in it. I think the two [religiosity and CVH] are very closely linked. I suspect a greater proportion of people from BAME backgrounds have some sense of faith and they are guided and led by a faith leader who will direct them to do or not do things (INT8, psychologist).

This participant emphasizes, in the above quote, the importance of religious beliefs in the UKEM community. She explains the important role of religious leaders in decision-making such as on COVID-19 vaccines among UKEM persons. This observation is consistent with evidence from a similar UK study (Woodhead et al., 2021), and UK evidence of a positive association between religiosity and CVH (Murphy et al., 2021).

#### Conspiracy theories

Higher levels of conspiracy and paranoiac beliefs has been associated with higher CVH (Freeman et al., 2020; Aw et al., 2021; Eberhardt and Ling, 2021a,b; Murphy et al., 2021). In line with this evidence, participants described conspiracy theories as a major factor in CVH: “And of course, behind all of this [CVH] there were also conspiracy theories. There was a lot of fake news flying around” (INT4, doctor). Several participants described the conspiracy theories they had encountered. For instance:

So there was all that [conspiracy theories] going around. So there’s some people saying the thing about religion. And then there were others saying things like, uhm, they’re trying to make us white…Why are they targeting the weak?…They cost the NHS too much, you know. All these older people they want to get rid of them…You know it’s really difficult not to get paranoid for a little while and think maybe there’s some truth in this (INT1, administrator).

INT1 narrates COVID-19 conspiracy theories about sacrilege, racial transformation and social Darwinism, and her uneasiness about these conspiracy beliefs. Other participants described their concerns about the conspiracy beliefs they had encountered:

Honest to God, I have heated argument with them [exponents]…because what they’re saying to me logically doesn’t make sense…It’s like you know, the conspiracy theory things that doesn’t go into your brain…So yeah, I really find it really really difficult (INT7, doctor).

The above narratives, and those shared by other participants, are similar to narratives from a study of residents of Bradford, UK (Lockyer et al., 2021) and from a systematic metasynthesis of COVID-19 conspiracy beliefs (van Mulukom et al., 2022). Some participants explained that they found the conspiracy narratives surprising and sometimes argued with or avoided engaging exponents in arguments as in similar UK studies (Lockyer et al., 2021; Woodhead et al., 2021).

#### Precipitancy and novelty

A major factor in vaccine hesitancy in general is concern about lack of rigorous testing of vaccines (European Centre for Disease Prevention and Control, 2015a; Karafillakis et al., 2016). Similarly, the hasty development and novelty of the COVID-19 vaccine has been implicated in CVH (Scientific Advisory Group for Emergencies, Ethnicity Sub-Group, 2020; Eberhardt and Ling, 2021a; Lockyer et al., 2021). Indeed, in affluent countries, increased concerns about rapid development of COVID-19 vaccines are associated with high CVH (Aw et al., 2021). This also emerged in the present study as explained by one participant:

I think I can totally understand why there would be hesitancy. I mean, yes, there is the fact that the vaccine was sort of made really quickly and every other vaccine was not made as quickly…Then you know, disaster could have arisen even more so (FGP3, nurse).

In the above quote, FGP3 explains that a factor in CVH is the fact that the COVID-19 vaccines were developed relatively quickly therefore leading to concerns about potential harmful effects. Indeed, among UKEM groups including healthcare workers, the speedy development of the COVID-19 vaccines has generated suspicion bordering on vaccine novelty and the sacrifice of standard procedure for expediency (Kamal et al., 2021; Woodhead et al., 2021).

#### Complications/side effects

Related to concerns about the hasty development and novelty of the COVID-19 vaccines are concerns regarding post-vaccination complications or side effects following vaccination (Aw et al., 2021; Kamal et al., 2021; Lockyer et al., 2021). Participants implicated these concerns in CVH. For instance:

I: What do you think of complications among persons who are actually vaccinated?

INT8, psychologist: That’s [complications/side effects] really what you don’t want, isn’t it? That’s enough to people who’re already hesitant. They’re definitely not going to take the vaccine.

The participant describes how post-vaccination complications/side effects reinforce CVH in line with literature on vaccine hesitancy in general (European Centre for Disease Prevention and Control, 2015a,b; Karafillakis et al., 2016; Acharya et al., 2021) and CVH in particular (Bogart et al., 2021; Deal et al., 2021; Kamal et al., 2021; Lockyer et al., 2021; Woodhead et al., 2021).

#### False positive test results

There is concern regarding the incidence of false negative and particularly false positive results from COVID-19 testing (Mayers and Baker, 2020; Surkova et al., 2020; Healy et al., 2021). Compared to false negative COVID-19 test results, false positive results are uncommon and not often discussed (Healy et al., 2021). One participant described the role of a false positive COVID-19 test result in generating mistrust and CVH in her family.

I went to get my test but the queue was so long that I didn’t bother going [to get tested]. But then they gave me a positive result. But I never even did the test …But you can see that by one thing going wrong, it takes you 10 20 steps backwards again with the family. So that was a difficult time (INT1, administrator).

As a background, INT1 had CVH challenges in her family and had been persuading her family members, particularly the husband, to get vaccinated. In the above quote, she explains that she received a positive COVID-19 test result despite not taking the test. She points out how such false positive test results have the potential to roll back efforts to mitigate CVH. It is estimated that the incidence of false positive results in PCR testing for COVID-19 is as high as 16.7% (Cohen and Kessel, 2020). False positive results can be caused by data and software issues, sample mislabeling, contamination, and low-level reactions in the PCR process (Cohen and Kessel, 2020; Surkova et al., 2020; Healy et al., 2021). Aside the potential domino effect of vaccine hesitancy in a person’s social network from false positive results as narrated by the participant above, false positive results have adverse physical and psychosocial effects.

These include an increased risk of exposure to COVID-19 inpatients and unnecessary treatment, needless isolation and contact tracing, psychological distress, and stigmatization (Surkova et al., 2020; Youmbi, 2020; Healy et al., 2021; Roy, 2021). For healthcare workers, false positive test results can be a huge incapacitation to themselves and the healthcare system (Surkova et al., 2020). Measures for minimizing the incidence of false positive results and alleviating adverse effects have been explored elsewhere (Mayers and Baker, 2020; Surkova et al., 2020; Healy et al., 2021; Layfield et al., 2021).

#### Social media and social support/influence

The proliferation of social media has facilitated information dissemination including false information. In this regard, social media is an important factor for disseminating false COVID-19 information and reinforcing CVH (Aw et al., 2021; Kamal et al., 2021). In line with the above, participants pointed to social media as a key platform for disseminating false COVID-19 information and mistrust thereby deepening CVH. For instance:

And the rest of the people that didn’t understand it [COVID-19 vaccination] and had read something on YouTube or Tik Tok or whatever social media that they have…And I believe always Facebook was a devil and I think I’m right. So all these things that people were getting [on social media], they were actually believing it. And I found it a challenge. So even on WhatsApp groups, like family groups or friends’ groups and all these groups, everybody is posting all this negativity (INT1, administrator).

As with the role of social media, participants underlined the important role of social support or influence in CVH. One participant noted that:

It also depends on each individual’s background and it is dependent on your network as well: who you were associated with and what kind of messages they were forwarding to you. If you were forwarded a lot of conspiracy theories, then you might end up being a bit too hesitant about the vaccine, whereas if you were surrounded by people who were much more encouraging of the vaccine, then you might not be that hesitant (INT4, doctor).

Similarly, one participant described side effects as having “a ripple effect through the community” (Joint interviewee [JINT] 2, doctor). The above perspectives reflect evidence that previous negative encounters of individuals, and their family and friends with formal services, and social media information reinforce CVH in UKEM groups (Kamal et al., 2021; Woodhead et al., 2021). Similarly, among US Black communities, pressure from significant others such as family and friends has been found as a reinforcer of COVID-19 vaccine acceptance (Bogart et al., 2021).

### Mitigating

Participants provided suggestions for mitigating CVH or improving COVID-19 vaccine uptake in the UKEM community. These are presented in the mitigating theme comprising the following subthemes: combating dis/misinformation and educating, non-judgmental approach, healthcare worker endorsement, community-oriented approach, convenience and self-determination, and gradual acceptance *via* risk–benefit self-assessment.

#### Combating dis/misinformation and educating

Combating dis/misinformation and educational programs are key to mitigating CVH (Roozenbeek et al., 2020; Acharya et al., 2021; Lockyer et al., 2021; Loomba et al., 2021; Murphy et al., 2021; World Health Organization and United Nations Children's Fund, 2021). Participants pointed out the importance of dealing with dis/misinformation and providing education on COVID-19. One participant noted that:

The solution might be essentially improving our healthcare knowledge and also limiting the false information spread over the Internet…There is sometimes false religious information and [information] that there are some other problems, or the vaccines are incompatible with some of the religions. I’m not clear whether it might work but sometimes I think policymakers should limit that kind of information or the spread of false information (INT2, doctor).

In addition to combating dis/misinformation, participants also indicated the importance of providing vaccine education in mitigating CVH. In the focus group, a participant (FGP3, nurse) narrated her experience as an immunization nurse in Australia. She explained that in the rollout of a vaccination programme for firefighters, healthcare workers persuaded hesitant firefighters to accept vaccination by providing them empirical evidence and reassurance on the benefits of vaccination.

Participants also noted the importance of religious education in mitigating CVH. Several exemplified the role of religious leaders in providing religious education and alleviating religious apprehension about COVID-19 vaccination. One interviewee noted:

I have to actually give praise to the scholars of all religions but especially the Muslims because at the beginning of it, they said this doesn’t have [pork]…So straight away, the religion scholars said it in the mosque - there is no pork in any of the vaccines so please when you’re offered it, have it. So that was good (INT7, doctor).

This perspective was corroborated in the joint interview:

I think a lot of that [religiosity] has been alleviated perhaps by religious leaders coming out and being very public about having the vaccine on social media and so on. There was like a drive-through NHS and other media platforms to sort of alleviate the fears of religious communities. So there was quite a lot of work done on that recently here in England and in the UK (JINT1, chaplain).

In the quotes presented above, participants commend and acknowledge the role of religious authorities in educational interventions addressing CVH in religious communities. The internet, particularly social media, is a major platform for the anti-vaccination movement and plays an prominent role in the spread of vaccine hesitancy (Dubé et al., 2015) and CVH in particular (Puri et al., 2020; Aw et al., 2021; Kamal et al., 2021; Petousis-Harris and Chan, 2021). Monitoring online pages and groups dedicated to vaccination refusal may elucidate dis/misinformation that can be addressed in educational interventions. Additionally, online methods such as search engine optimization, fact-checking, and blockchain technology (Khurshid, 2020; Desai, 2021) may facilitate the combating of dis/misinformation and the streamlining and visibility of educational material targeted at hesitant persons.

This is important with evidence that dis/misinformation and related factors such as information overload, complexity, and contradiction generate mistrust and contribute to CVH among UKEM groups (Kamal et al., 2021). Education about the vaccines such as ingredients, safety, effectiveness, side effects, and the robustness of the vaccine trials may be useful in mitigating CVH (Bogart et al., 2021; Kamal et al., 2021; World Health Organization and United Nations Children's Fund, 2021; Iyengar et al., 2022).

Also, monitoring and reporting post-vaccination side effects may provide useful information to the community and provide reassurance of vaccine efficacy in dealing with dis/misinformation (Petousis-Harris and Chan, 2021; Reid and Mabhala, 2021; Iyengar et al., 2022). In a study of 32 migrants in the UK, they indicated the lack of COVID-19 information in language they understand (Deal et al., 2021). Targeted multilingual information may therefore be useful in mitigating CVH in migrant populations (Iyengar et al., 2022). Although religious leaders and organizations may not be trusted as a source of medical information (Bogart et al., 2021), they can be useful in alleviating religious concerns about COVID-19 vaccination thereby helping in the mitigation of CVH in the UKEM community as pointed out above by participants (INT7, doctor; JINT1, chaplain; JINT2, doctor).

#### Non-judgmental approach

A non-judgmental approach to healthcare connotes openness and agreeableness toward clients and patients despite differences in health status, background and personal experience, culture, and perspective (Branson et al., 2022). This approach is crucial for effective healthcare (Koh, 1999; Branson et al., 2022). It emerged that a non-judgmental approach is important in boosting COVID-19 vaccine confidence. Two focus group participants emphasized this:

FGP1, doctor: I think listening [to hesitant persons is important]…and like have that conversation…You just need to have a conversation with people. And sometimes that’s all it needs.

FGP3, nurse: I think as you say that [FGP1, doctor], a non-judgmental approach is definitely what’s needed.

FGP1, doctor: That’s what I mean. A non-judgmental approach.

FGP3, nurse: Yeah…And just a reassurance for patients and staff as well.

In the above quote, discussants agree on the importance of a non-judgmental approach in persuading COVID-19 vaccine-hesitant persons to accept vaccination. It is noteworthy that targeted interventions may generate reactance from healthcare workers and the UKEM community due to perceived stigmatization thereby having a counterproductive effect (Healthwatch, 2021a; Millward, 2021; Woodhead et al., 2021). This underlines the importance of a non-judgmental approach to intervention design and rollout. We also noted previously that mistrust associated with CVH in the UKEM community has roots in history and religion, conspiracy theories, vaccine “novelty,” side effects among others. Allaying these suspicions and misgivings through a non-judgmental approach has been demonstrated to be effective in mitigating CVH (Breckenridge et al., 2021).

Aside combating dis/misinformation and educating as noted previously, elucidating the emotional manipulation tactics used against vaccination, dispelling negative emotions (e.g., fear and apprehension), and fostering positive emotions (e.g., altruism and utilitarianism) in COVID-19 vaccination interventions is important for mitigating CVH (Chou and Budenz, 2020; Freeman et al., 2020; Petousis-Harris and Chan, 2021). Additionally, appreciating the historical as well as socioeconomic inequalities that underpin CVH is important for designing interventions targeted at mitigating CVH in the UKEM community (Jaiswal et al., 2020; Scientific Advisory Group for Emergencies, Ethnicity Sub-Group, 2020; Bogart et al., 2021; Woodhead et al., 2021). Moreover, with evidence that undocumented migrants in the UK harbor fear about immigration checks at COVID-19 vaccination centers despite government assurance to the contrary (Deal et al., 2021), the importance of “reassurance” (FGP3, nurse) in mitigating CVH cannot be underestimated.

#### Healthcare worker endorsement

Healthcare workers advocating for or promoting a health intervention such as a vaccine to their client or patient is a major factor in vaccine uptake (European Centre for Disease Prevention and Control, 2015a; Dubé et al., 2018; Olanipekun et al., 2020). Several participants indicated that healthcare worker endorsement of COVID-19 vaccines is a persuasive approach to mitigating CVH. A participant described her observation of CVH in the UKEM community and her persuasive role using endorsement as a healthcare worker:

From the figures that I could see very clearly [from my senior management position], BAME people did not want the vaccine. And we had all these focus groups set up, and we had these BAME networks setup. And obviously, I was running into some of them just to understand what the issue is…I was giving my own example in the chat to say…I’ve already had the vaccine…when you’re offered it, take it (INT1, administrator).

Here, the participant indicated being clinically vulnerable and her willingness to be transparent about this and accepting vaccination. This was her assurance and encouragement to COVID-19 hesitant patients on the importance of accepting COVID-19 vaccination. The above narrative reflects evidence showing that healthcare worker endorsement is important in mitigating CVH (Freeman et al., 2020; Bogart et al., 2021; Cambon et al., 2022). Indeed, lack of physician advocacy tends to increase CVH in affluent countries (Aw et al., 2021). Healthcare workers such as doctors and nurses are credible and trusted sources of COVID-19 information (Bogart et al., 2021; World Health Organization and United Nations Children's Fund, 2021). Accordingly, the incorporation of UKEM healthcare worker endorsement in community, social media, and telehealth campaigns can be useful in mitigating CVH in the UKEM community (Puri et al., 2020; Scientific Advisory Group for Emergencies, Ethnicity Sub-Group, 2020; Acharya et al., 2021; Bogart et al., 2021; World Health Organization and United Nations Children's Fund, 2021; Malhotra et al., 2022).

#### Community-oriented approach

Communities are crucial to health promotion (Jagosh et al., 2015; Castillo et al., 2019; Meyer et al., 2021). In line with the above, participants pointed out the importance of a community-oriented approach in mitigating CVH:

They did quite a few vaccine clinics in east, and here in Bristol in some local mosques. And that worked really well. And that’s as [JINT1, chaplain] pointed out, going to where people would go. And it removes some element of distrust, I think (JINT2, doctor).

The other joint interviewee indicated:

So they [religious leaders] are crucial. Which is why you know when it comes to even encouraging vaccines in these communities, you have to go to these places: the mosques, the temples, the churches and stuff. Because that’s how you’re gonna reach the community. And that’s how you’re going to change their mind (JINT1, chaplain).

These participants highlight, in the quotes supra, the importance of community collaboration on vaccine uptake with trusted leaders in familiar and accessible venues. It has been demonstrated that fostering COVID-19 vaccination utilitarianism is associated with decreased CVH (Freeman et al., 2020). As advocated by participants above, UKEM community engagement, intervention co-creation, partnerships and investment is important in the mitigation of CVH among members of the UKEM community (Rose et al., 2020; Warren et al., 2020; Acharya et al., 2021; Bogart et al., 2021; Deal et al., 2021; Iyengar et al., 2022).

#### Convenience and self-determination

Aside the psychosocial and communication factors discussed above, convenience in terms of practicality, availability, and accessibility is a major factor in vaccine (European Centre for Disease Prevention and Control, 2015b) and COVID-19 vaccine (Aw et al., 2021; Healthwatch, 2021b) uptake. One participant noted:

You know, make it accessible [so] the people can fit it in…Access, access is really important because if you’re working everyday Monday to Friday, how are you going to fit in a vaccine? You can’t make the time for a vaccine, right? Yeah, it has to be accessible (JINT1, chaplain).

In the above quote, the participant points out the importance of convenience in COVID-19 vaccine uptake. We previously discussed the importance of socioeconomic considerations in the risk–benefit self-assessment of accepting COVID-19 vaccines. It is therefore important that the economic anxiety emanating from COVID-19 (Bareket-Bojmel et al., 2021) particularly in the UKEM community (Rose et al., 2020; Murphy et al., 2021) is taken into consideration in designing interventions addressing CVH in the UKEM community (Jaiswal et al., 2020). Extending and making flexible vaccination hours and diversifying vaccination points (e.g., community centers, foodbanks and charities, pharmacies, and pop-up venues) can enhance convenience and increase COVID-19 vaccine uptake in UKEM communities (Scientific Advisory Group for Emergencies, Ethnicity Sub-Group, 2020; Bogart et al., 2021; Deal et al., 2021).

In line with convenience, self-determination theory (Ryan and Deci, 2000) underscores the importance of autonomous motivation in health behavior such as vaccination (Fall et al., 2018). Ideally, individuals should have the prerogative of choosing among alternative treatments (Hughes et al., 2021). Given the variety of vaccines available, another participant emphasized self-determination in mitigating CVH:

People should have an option of what vaccination they will get if they are afraid of what is being put out there…I think there should be an option for people to say: ok if I have to, I don’t want this; I would like to take this one. Because if you want people to do something, then you should have facilities to encourage them to do it (INT3, nurse).

Experimental evidence from the UK (McPhedran and Toombs, 2021) and a survey of a nationally representative Hungarian sample (Kutasi et al., 2022) suggest different preferences for COVID-19 vaccines. Indeed, COVID-19 vaccine characteristics, particularly their level of efficacy, are critical to their acceptance in the UK (McPhedran and Toombs, 2021). Consistent with self-determination theory, additional experimental evidence from Germany shows that with the availability of multiple COVID-19 vaccines, restriction of vaccine choice evokes anger, and that allocation of one’s preferred vaccine reduces CVH, whereas CVH increases when the non-preferred vaccine is allocated (Sprengholz et al., 2021). In sum, the above studies provide empirical support for the importance of convenience (Scientific Advisory Group for Emergencies, Ethnicity Sub-Group, 2020; Aw et al., 2021; Healthwatch, 2021a; World Health Organization and United Nations Children's Fund, 2021) and self-determination (Dal-Ré et al., 2021; Hughes et al., 2021) in mitigating CVH.

#### Gradual acceptance *via* risk–benefit self-assessment

The vaccine hesitancy model (Peretti-Watel et al., 2015) posits vaccine hesitancy as a decision-making process involving a negotiation of trust in health/medical authority versus personal health/risk. Considering the above-noted mistrust, some participants explained how they gradually overcame their CVH and accepted vaccination as in a similar UK study (Woodhead et al., 2021). One participant indicated that:

There are always risks to every single medicine or vaccination that we take. I was offered the vaccine probably quite early on, probably like January. And it took me until beginning of March to really say: yes, I put my arm out. And even then, I was really cautious and not sure. So I didn’t go running to be jabbed (INT8, psychologist).

Other participants provided more-detailed descriptions of the important role of risk–benefit self-assessment (Duong et al, 2021) in overcoming CVH and accepting vaccination. One participant explained:

[that people’s lives are] already under a lot of strain I suppose. So by taking the vaccine, maybe it appears to them to be taking a risk. They’re going to become possibly unwell through it, through an unknown side effect possibly. Even the fact that they have to take a few days off to recover; if they’re working on a contract which doesn’t allow them to be sick, it doesn’t pay them to be sick, right? Then you got issues there about: can you afford to stay at home? What will taking the vaccine; what kind of consequence will that have on your income, on your family, on your work? (JINT1, chaplain).

This participant describes the health and socioeconomic considerations and the risk–benefit self-assessment of accepting COVID-19 vaccines. This perspective is reasonable given evidence that persons of UKEM background tend to have lower income as well as the inverse association of income with CVH (Murphy et al., 2021) and COVID-19 mortality (Rose et al., 2020) in the UKEM community. Indeed, it has been demonstrated in a study of the UK, USA, and Israel that people in these countries have a comparable level of anxiety about the economic effects of COVID as they do about the health effects (Bareket-Bojmel et al., 2021) therefore transposing COVID-19 as an “economic pandemic.” In short, in designing interventions targeted at mitigating CVH in the UKEM community, consideration must be given to the present finding that overcoming CVH and accepting COVID-19 vaccination is not an event but a psychological negotiation process of trust in health/medical authority versus personal health/risk as proposed in the vaccine hesitancy model (Peretti-Watel et al., 2015). The uptake benefits of such interventions may not be evident in the short-term but in the long-term.

### Strengths, limitations, and future directions

As far as we are aware, the present study is the first to explore UKEM healthcare workers’ experiences of CVH in the UKEM community. Based on the sentinel perspective of our sample of UKEM healthcare workers, our study elucidates the CVH phenomenon in the UKEM community, important ingredients in interventions addressing CVH and boosting COVID-19 vaccine uptake and directions for COVID-19-related clinical practice in the UKEM community. The mixed qualitative design, comprising individual and joint interviews as well as a focus group is desirable and provides a method triangulation advantage (Carter et al., 2014) as noted previously and is therefore another strength of our study. Also, the demographic and profession diversity of our UKEM sample is advantageous in the exploration of a diversified UKEM healthcare worker perspective. OD’s prior working relationships with three of the participants facilitated their recruitment and may have contributed to their availability for interviews (Deakin and Wakefield, 2014). To avert any interviewer bias, “familiar interviews” were led by DS who had no previous working relationship or acquaintanceship with participants. It is our opinion that familiarity facilitated rapport and did not negatively affect participants’ responses (Rodriguez et al., 2015; Weinreb et al., 2018; Roiha and Iikkanen, 2022).

Still, sample non-representativeness implies our findings may not be generalizable to the UKEM healthcare worker population, White British/Irish healthcare workers, and the general lay population. It is noteworthy, however, that similar perspectives and evidence have been reported in previous healthcare worker and general population/community studies in the UK (Kamal et al., 2021; Woodhead et al., 2021) and other countries (Aw et al., 2021; Paul et al., 2022). Nonetheless, future studies are encouraged to use representative samples to enhance the external validity of findings. Moreover, our qualitative design does not permit the exploration of the prevalence of perspectives expressed by our sample in the UKEM community. Future studies of representative UKEM healthcare worker and community samples may provide, among others, evidence of the prevalence of CVH perspectives.

Also, the perspectives expressed in the present study should be interpreted considering the preceding rollout of COVID-19 vaccines in the UK. Similarly, the experiences of our COVID-19-vaccinated UKEM healthcare workers may implicitly not be generalizable to CVH healthcare workers. For comprehensive evidence of CVH, it is critical that the perspectives of unvaccinated healthcare workers are explored in future studies. Furthermore, the use of “BAME” in participants’ quotes should be treated with caution and interpreted as partly reflecting interviewer priming. It came to our attention, during the manuscript review process, that the terms “BAME” (black, Asian and minority ethnic) and “BME” (black and minority ethnic) have been criticized by UK government as exclusionary, lacking nuance, and misleading (GOV.UK, 2021). It is notable that some participants, such as INT2 and FGP1 in their quotes presented herein under the Heterogeneity theme, seem to point to this although not explicitly. As justified supra, we replaced “BAME” with “UKEM” in the present paper in line with UK government guidelines.

## Conclusion

The perspectives of UKEM healthcare workers are critical to dealing with the disproportionate prevalence of CVH in the UKEM community. From the experiences of our sample of UKEM healthcare workers, CVH in the UKEM community is a multifaceted issue intersecting personal (educational attainment and ethnicity), social (conspiracy theories, social media and social support/influence), institutional (historical medical malpractice and religion), and public policy (vaccine precipitancy and novelty, vaccine complications/side effects, and false positive test results) factors. Accordingly, interventions targeted at mitigating CVH and increasing COVID-19 vaccine uptake in the UKEM community should adopt a multicomponent strategy. Specifically, they should be non-judgmental, combat dis/misinformation and educate, include UKEM healthcare worker endorsement, be community-oriented, enhance the convenience of vaccination centers, consider the possibility of vaccine choice, and appreciate the gradual process and risk–benefit assessment in overcoming CVH and accepting vaccination.

## Data availability statement

The raw data supporting the conclusions of this article will be made available by the authors, without undue reservation.

## Ethics statement

The studies involving human participants were reviewed and approved by the Faculty Research Ethics Committee of the Faculty of Health and Life Sciences, De Montfort University (HLS FREC Ref: 3662). Participants provided their written informed consent to participate in this study. The patients/participants provided their written informed consent to participate in this study.

## Author contributions

DS, CO, and OD designed the study. DS, ZA, and OD collected the data. DS and PA conducted the data coding and transcription. DS conducted the data analysis. All authors contributed to the article and approved the submitted version.

## Conflict of interest

The authors declare that the research was conducted in the absence of any commercial or financial relationships that could be construed as a potential conflict of interest.

## Publisher’s note

All claims expressed in this article are solely those of the authors and do not necessarily represent those of their affiliated organizations, or those of the publisher, the editors and the reviewers. Any product that may be evaluated in this article, or claim that may be made by its manufacturer, is not guaranteed or endorsed by the publisher.
